# Integrated Nutrient Management for Rice Yield, Soil Fertility, and Carbon Sequestration

**DOI:** 10.3390/plants11010138

**Published:** 2022-01-05

**Authors:** Tahmina Akter Urmi, Md. Mizanur Rahman, Md. Moshiul Islam, Md. Ariful Islam, Nilufar Akhtar Jahan, Md. Abdul Baset Mia, Sohela Akhter, Manzer H. Siddiqui, Hazem M. Kalaji

**Affiliations:** 1 Department of Soil Science, Faculty of Agriculture, Bangabandhu Sheikh Mujibur Rahman Agricultural University, Gazipur 1706, Bangladesh; urmi.bsmrau@gmail.com (T.A.U.); mizan@bsmrau.edu.bd (M.M.R.); 2 Department of Agronomy, Faculty of Agriculture, Bangabandhu Sheikh Mujibur Rahman Agricultural University, Gazipur 1706, Bangladesh; 3 Department of Agriculture, Bangabandhu Sheikh Mujibur Rahman Science and Technology University, Gopalganj 8100, Bangladesh; arifulislam@bsmrstu.edu.bd (M.A.I.); nilufarjahan@bsmrstu.edu.bd (N.A.J.); 4 Department of Crop Botany, Faculty of Agriculture, Bangabandhu Sheikh Mujibur Rahman Agricultural University, Gazipur 1706, Bangladesh; miabaset@bsmrau.edu.bd; 5 Soil Science Division, Bangladesh Agricultural Research Institute, Gazipur 1701, Bangladesh; sohela_akhter@yahoo.com; 6 Department of Botany and Microbiology, College of Science, King Saud University, Riyadh 11451, Saudi Arabia; mhsiddiqui@ksu.edu.sa; 7 Department of Plant Physiology, Institute of Biology, Warsaw University of Life Sciences SGGW, 159 Nowoursynowska 159, 02-776 Warsaw, Poland; hazem@kalaji.pl; 8 Institute of Technology and Life Sciences, National Research Institute, Falenty, Al. Hrabska 3, 05-090 Raszyn, Poland

**Keywords:** carbon sequestration, nutrient-use efficiency, poultry manure, rice yield, soil fertility, vermicompost

## Abstract

Reliance on inorganic fertilizers with less or no use of organic fertilizers has impaired the productivity of soils worldwide. Therefore, the present study was conducted to quantify the effects of integrated nutrient management on rice yield, nutrient use efficiency, soil fertility, and carbon (C) sequestration in cultivated land. The experiment was designed with seven treatments comprising of a zero input control, recommended inorganic fertilizers (RD), poultry manure (PM) (5 t ha^−1^) + 50% RD, PM (2.5 t ha^−1^) + 75% RD, vermicompost (VC) (5 t ha^−1^) + 50% RD, VC (2.5 t ha^−1^) + 75% RD, and farmers’ practice (FP) with three replications that were laid out in a randomized complete block design. The highest grain yield (6.16–6.27 t ha^−1^) was attained when VC and PM were applied at the rate of 2.5 t ha^−1^ along with 75% RD. Uptake of nutrients and their subsequent use efficiencies appeared higher and satisfactory from the combined application of organic and inorganic fertilizers. The addition of organic fertilizer significantly influenced the organic carbon, total carbon, total nitrogen, ammonium nitrogen, nitrate nitrogen, soil pH, phosphorus, potassium, sulfur, calcium, and magnesium contents in post-harvest soil, which indicated enhancement of soil fertility. The maximum value of the organic carbon stock (18.70 t ha^−1^), total carbon stock (20.81 t ha^−1^), and organic carbon sequestration (1.75 t ha^−1^) was observed in poultry manure at the rate of 5 t ha^−1^ with 50% RD. The soil bulk density decreased slightly more than that of the control, which indicated the improvement of the physical properties of soil using organic manures. Therefore, regular nourishment of soil with organic and inorganic fertilizers might help rejuvenate the soils and ensure agricultural sustainability.

## 1. Introduction

Globally, agriculture is challenging because of climate change and soil health degradation. Organic matter (OM) is a vital component to keep soil alive and productive, and for providing better ecosystem services [1,2]. The importance of soil OM is further revealed through the “4 per 1000 initiative’’ at the COP21 Paris climate summit in 2015. Even though crop production has increased many fold since the last 50-years, crop yields still need to be increased to meet the food requirements of an ever-burgeoning global population. Along with global agriculture, Bangladesh also faces the challenge of producing crops from its limited land resources to meet the huge demand from its population. The scope is limited to bring new land under cultivation. Moreover, every year, cultivated land is being reduced due to human settlement and rapid urbanization and industrialization. Therefore, the intensification of land with modern crop varieties for increased production is crucial [3]. Rice is the staple food for the 170 million people of the country and is grown on around 10.5 million ha [4]. The global annual production of rice is about 496.40 million tons from 162.06 million hectares of land in 2019–2020 [5]. Although modern rice varieties have the highest yield, there is still a serious yield gap. In Bangladesh, the lack of judicial nutrient management is considered as the major causes of yield gap in rice production. An imbalanced use of inorganic fertilizers and pesticides without organic fertilizers has led to deterioration of soil health and crop yield loss which has become a concern. More dependency on chemical fertilizers and imbalanced nutrient management practices has impaired the productivity of soils in many Asian countries including Bangladesh. Increased the cropping intensity and regular cultivation of high-yielding rice varieties has increased the removal of nitrogen (N), phosphorus (P), potassium (K), and other macro- and micro-nutrients from the soils in Bangladesh [6].

Soil OM content is reported to be declining, which is considered as one of the most serious threats in Bangladesh agriculture [3]. The integration of all organic, natural, and inorganic sources of nutrients is an efficient and environmentally friendly technology of crop production which is known as integrated nutrient management (INM). Management practices, such as diversified cropping systems and the application of different organic wastes and optimum fertilization, are believed to offer high potential for increasing the nutrient-use efficiency, carbon © levels in soils, and the crop yield [3,7,8,9]. In addition, the release pattern of inorganic fertilizers compared to organic ones are higher [3]. As a result, the released nutrients are used and lost rapidly by different means. On the other hand, organic fertilizers are decomposed slowly and nutrients are available for a longer period of time which helps to maintain soil nutrient status. However, most of the soils of Bangladesh have an OM content that is less than 1.5% and, in many cases, it is less than 1% [10]. Hence, a large amount of N fertilizer is used to increase soil fertility and crop productivity. Excess N may enhance mineralization of OM, which may decrease soil C content and increase in carbon dioxide (CO_2_) emission. If the present rate of its degradation is continued, in the near future, the soil would become barren. Poultry manure (PM) and vermicompost (VC) are the potential organic sources of soil organic C and plant nutrients [11,12]. Many reports showed that PM and VC are the source of N, P, K, Ca, Mg, and S that can improve the soil fertility and the grain crops can uptake more of these nutrients [3,11,13]. As VC is rich in microbial activity and contains antagonistic organisms to control plant pathogens, it is also, therefore, an effective biocontrol agent [14]. Jorgensen et al. [15] reported that adding PM and VC in particular increases the contents of organic carbon (OC) and of microbial biomass, the cation exchange capacity, and the biological activities of soils. The above findings suggests that organic matter along with inorganic fertilizers can improve soil fertility as well as soil organic C than when applied alone.

Carbon sequestration is vital for sustaining ecosystem productivity and reducing greenhouse gas emissions in the context of global warming and climate change [16]. Carbon sequestration can develop soil structure, enrich microbial diversity, and improve soil health. Soil has the potential to sequester 3.3 times more C than that of the atmospheric C pool [17]. Soil can act as a sink or source of atmospheric CO_2_ depending on the soil and crop management practices and climatic conditions. The soils of Bangladesh have low reserves of C and plant nutrients due to increasing cropping intensity, higher rates of decomposition of organic matter under prevailing hot and humid climate, use of lesser quantity of organic manure, and little or no use of green manure [3]. It is predicted that the crop production of Bangladesh would be tremendously vulnerable to climate change and, therefore, the food security will be at risk. The addition of C-enriched materials such as PM and VC improves soil physical, chemical, and biological characteristics. The improvement of soil properties favors the development of the crop root system, elongating it both at the surface level and in the deep soil, which ultimately helps to accumulate more C in the soil. The continuous application of organic manure builds up the soil C content and ensures an adequate N supply. The soil organic C pool in agricultural land is capable of enhancing agricultural sustainability and serving as a potential sink of atmospheric CO_2_ [18]. The global concerns about the effect of climate change demand intensive research on carbon cycling, its transformation under different crops and soil management practices, and the stability of C compounds in soils [19]. Proper management of organic manures and wastes/compost, reduced tillage, improving soil biodiversity, micro-aggregation, and mulching can play an important role in reducing CO_2_ emission and increase the C sequestration in soil [20]. However, it is uncertain whether crop fields accumulate C and how organic carbon (OC) level changes with different management practices, and so need to be investigated. Up-to-date information on the dynamics of soil C under different soil and crop management practices is necessary to help to maintain a good level of carbon for soil health and its productivity and restraining from global warming. The present study was aimed to compensate for the lack of such studies on C sequestration and soil fertility under different organic and inorganic fertilizer management options in rice cultivation at the farmer’s field level. Therefore, the objectives of the study were to quantify the effects of integrated nutrient management on rice yield to determine recovery, agronomic, and physiological efficiencies of nutrient use in rice cultivation as affected by integrated nutrient management, and to assess soil fertility and determine the rates of C sequestration under different integrated nutrient management practices.

## 2. Results

### 2.1. Rice Yields

The grain yield of rice was significantly influenced by different treatments and all the treatments significantly gave a higher grain yield of rice over the control (Table 1). The application of poultry manure (PM) and vermicompost (VC) at the rate of 2.5 t ha^−1^ along with 75% of the recommended inorganic fertilizers (RD) produced the highest grain yield (6.16 and 6.27 t ha^−1^, respectively) (*p* < 0.05) even over the recommended doses of N, P, and K (5.33 t ha^−1^). However, the effects of these treatments in producing grain yield were insignificantly different with that of PM 5 t ha^−1^ along with 50% RD. The straw yield of rice was significantly influenced by the application of different treatment (Table 1). Statistically, the highest straw yield (7.35 t/ha) was found when VC was applied at the rate of 2.5 t ha^−1^ with 75% RD and a statistically similar straw yield (7.24 t/ha) was found when PM was applied at the rate of 2.5 t ha^−1^ with 75% RD. Therefore, the combination of both organic manures and inorganic fertilizer produced a better straw yield of rice. The highest biological yield (13.6 t/ha) was also obtained when VC was applied at the rate of 2.5 t ha^−1^ along with 75% RD and a statistically similar biological yield (13.4 t/ha) was found when PM was applied at the rate of 2.5 t ha^−1^ along with 75% RD.

### 2.2. Nutrient Uptake by Rice

**Figure 1:**
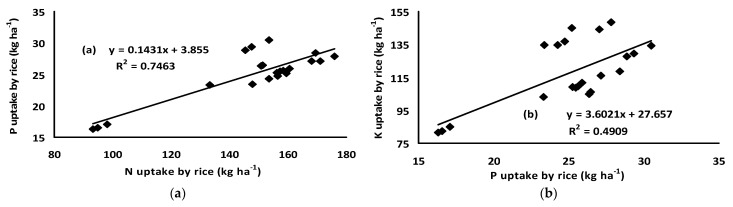

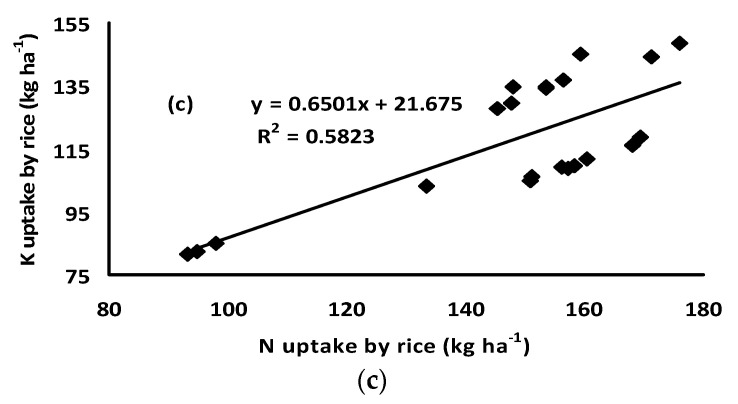
Relationship between the different nutrients uptake by rice: (**a**) N & P, (**b**) P & K, and (**c**) N & K uptake by rice.

**Figure 2:**
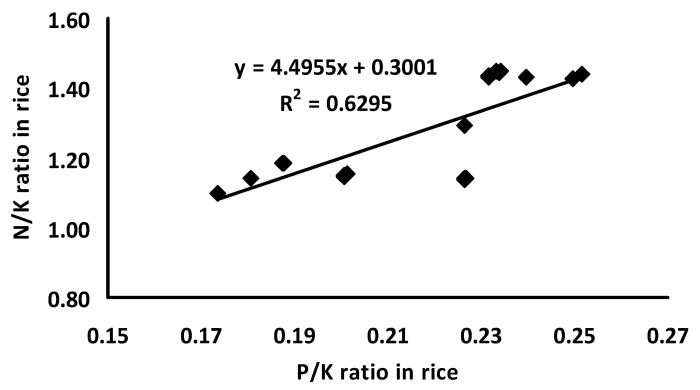
Relationship between the N/K and P/K ratios in rice plant.

The nutrient uptake by rice among different treatments varied significantly (Table 2). The treatment comprising of VC at the rate of 2.5 t ha^−1^ with 75% RD recorded the maximum nitrogen uptake in rice (168.86 kg/ha) whose effect was statistically identical to PM at the rate of 2.5 t ha^−1^ with 75% RD (166.06 kg/ha). The highest phosphorus uptake by rice (29.57 kg/ha) was observed in the treatment comprising of PM at the rate of 5 t ha^−1^ with 50% RD. The highest potassium uptake (145.97 kg/ha) was found in the treatment comprising of VC at the rate of 2.5 t ha^−1^ with 75% RD whose effect was statistically superior to the rest of the treatments. The second highest K uptake (135.46 kg/ha) was noted in the treatment comprising of VC at the rate of 5 t ha^−1^ with 50% RD. A significant linear relationship between the uptake of N–P, P–K, and N–K by rice was also observed (Figure 1). Moreover, the significant linear relationship between N:K and P:K in rice under different treatments showed that the treatments had a significant effect on the uptake of these nutrients (Figure 2).

### 2.3. Nutrient Use Efficiency

**Figure 3:**
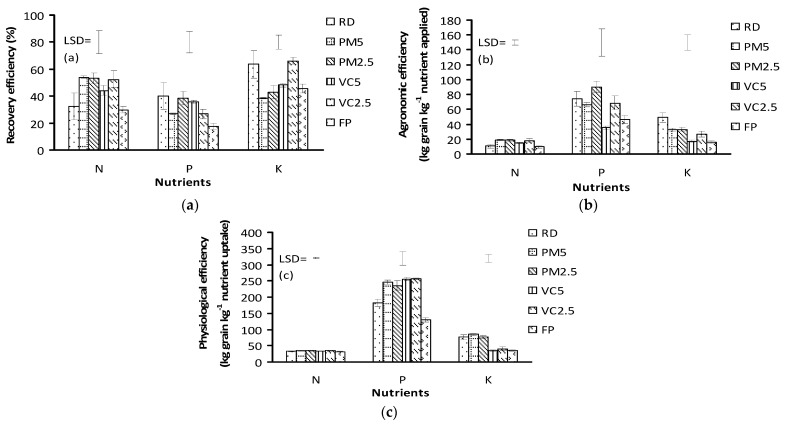
Nutrient use efficiencies (**a**) recovery efficiencies, (**b**) agronomic efficiencies, and (**c**) physiological efficiencies in the integrated nutrient management of organic and inorganic fertilizers in rice. Note: RD = recommended inorganic fertilizers; PM = poultry manure; VC = vermicompost; FP = farmer’s practice.

Recovery, agronomic, and physiological efficiency of N, P, and K showed significant differences among the treatments in rice cultivation (Figure 3). The recovery efficiency (RE) of nitrogen was found to be significantly higher in the treatments comprising of the organic and inorganic fertilizers (56–58%) over the RD and farmers practice (Figure 3a). The recovery efficiency of P was found to be significantly higher in the RD treatment (40%), which appeared statistically similar to the treatments comprising of PM 2.5 t ha^−1^ plus 75% RD (39%) and VC 5 t ha^−1^ plus 50% RD (36%). The recovery efficiencies of K were found to be significantly higher in the VC 2.5 t ha^−1^ plus 75% RD (66%) and RD (64%). The agronomic efficiency (AE) of nitrogen was found to be significantly higher in the treatments comprising of PM 5 t ha^−1^ plus 50% RD (19 kg grain kg^−1^ N applied), PM 2.5 t ha^−1^ plus 75% RD (18 kg grain kg^−1^ N applied), and VC 2.5 t ha^−1^ plus 75% RD (18 kg grain kg^−1^ N applied) and among these treatments, AE were not significantly different (Figure 3b). The AE of P was found to be significantly higher in the treatment comprising of PM 2.5 t ha^−1^ plus 75% RD, which was 90 kg grain kg^−1^ P applied. The AE of K was found to be significantly higher in the RD treatment (49 kg grain kg^−1^ N applied). However, poultry manure and vermi-compost contributed reasonably to the agronomic efficiency of potassium. The physiological efficiency (PE) of N and P in all the treatments was rational and acceptable, while PM and VC along with the inorganic fertilizers provided significantly higher efficiencies (35 kg grain kg^−1^ N uptake; 234–257 kg grain kg^−1^ P uptake, respectively) (Figure 3c). The PE of K was found to be significantly higher in the PM (77–85 kg grain kg^−1^ K uptake) and the RD (77 kg grain kg^−1^ K uptake) treatments.

### 2.4. Soil Organic Carbon and Total Carbon

The treatments did not show significant variation in the organic carbon contents in soils (Table 3). On the other hand, the total carbon contents significantly varied among the different treatments, but there was no variation among the organic treatments. However, compared to the control and RD treatments, small increases in organic and total carbon contents in soils were observed due to the addition of PM and VC at the rate of 5 t ha^−1^.

### 2.5. Total and Available Nitrogen Content in Soil

Significant effects of the different treatments were observed in the total and available nitrogen contents in soils (Table 3). The maximum total nitrogen content (0.124%) was observed in PM at the rate of 5 t ha^−1^ with 50% RD, which was statistically similar with VC at the rate of 5 t ha^−1^ with 50% (0.109%) and VC at the rate of 2.5 t ha^−1^ with 75% RD (0.099%). The maximum ammonium (9.29 mg kg^−1^) and nitrate nitrogen contents (3.55 mg kg^−1^) were observed in PM at the rate of 2.5 t ha^−1^ with 50% RD. The lowest total nitrogen content (0.073%) was found in the RD treatment. There was no statistically significant difference in the total N contents in the soils between the control (0.077%) and the RD (0.073%) treatments.

### 2.6. Physical and Chemical Properties of Post-Harvest Soils

Significant variations in the physical and chemical properties of soil under different treatments at crop harvest were observed (Table 4). The treatments’ effect on the bulk density was found inconsistent with the addition of organic fertilizers (Table 4). The bulk density was slightly lowered by the addition of organic materials. A significantly lower bulk density (1.18 g cm^−3^ ) of soil was observed in PM at the rate of 5 t ha^−1^ with 50% RD. The data revealed that soil pH was strongly acidic in the control treatment, while it increased to slightly acidic due to the addition of PM and VC to soils (Table 4). The highest soil pH (6.25) was observed in the PM at the rate of 5 t ha^−1^ with 50% RD which was statistically similar with VC at the rate of 5 t ha^−1^ with 50% RD. The treatments comprising of organic and inorganic fertilizers significantly increased the available phosphorus (P) contents in the soils over the control and farmers’ practice (*p* < 0.05) (Table 4). The available P in the soils of the control treatment was 2.69 mg kg^−1^. Due to the application of PM and VC at the rate of 5 t ha^−1^ , the available P contents were increased to 5.66 and 4.02 mg kg^−1^, respectively. The sulfur (S) content of the post-harvest soils ranged from 2.89 to 8.85 mg kg^−1^ (Table 4). The highest value of S (8.85 mg kg^−1^) was recorded in VC at the rate of 5 t ha^−1^ with 50% RD whose effect was not statistically similar with the other treatments. An appreciable amount of available S was increased by the application of organic matter in soil. The application of PM and VC caused a considerable amount of increase in the potassium (K) content over the initial value (Table 4). The potassium content was found to be significantly higher (0.33 c-mol kg^−1^ soil) when VC was applied at the rate of 5 t ha^−1^ with 50% RD. These results indicated that exchangeable K content was higher in the soils that were treated with organic manures than those treated with chemical fertilizers. The application of PM and VC caused a considerable increase in the calcium (Ca) content over the initial value (Table 4). The highest value of Ca (5.07 c-mol kg^−1^ soil) was observed in VC at the rate of 5 t ha^−1^ with 50% RD whose effect was statistically superior to the rest of the treatments. The second highest value (3.80 c-mol kg^−1^ soil) of Ca in the soils was found in PM at the rate of 5 t ha^−1^ with 50% RD. The treatments’ effect on the soil magnesium content (Mg) was found to be consistent with the addition of organic fertilizers (Table 4). The highest Mg content (1.14 c-mol kg^−1^ soils) was observed in VC at the rate of 2.5 t ha^−1^ with 75% RD whose effect was statistically superior to the rest of the treatments.

### 2.7. Soil Organic Carbon Stock, Total Carbon Stock, and Organic Carbon Sequestration in Post-Harvest Soil

The treatments did not show significant variation in the organic carbon stock, total carbon stock, and organic carbon sequestration in post-harvest soils (Table 5). Among the treatments, the highest value of organic carbon stock (18.70 t ha^−1^), total carbon stock (20.81 t ha^−1^), and organic carbon sequestration (1.75 t ha^−1^) was observed in PM at the rate of 5 t ha^−1^ with 50% RD and the second highest was observed in VC at the rate of 5 t ha^−1^ with 50% RD. The lowest value of organic carbon stock (17.44 t ha^−1^), total carbon stock (18.43 t ha^−1^), and organic carbon sequestration (0.49 t ha^−1^) was recorded in the control. The treatments that combined the organic amendments and inorganic fertilizers had greater SOC stock compared to those that received only inorganic fertilizers.

## 3. Discussion

### 3.1. Rice Yields

The highest grain yields were obtained when PM and VC were applied at the rate of 2.5 t ha^−1^ along with 75% recommended inorganic fertilizers (Table 1). Inorganic fertilizers supply nutrients immediately after application, while organic fertilizers release nutrients slowly through microbial mineralization which ensure that the nutrient availability in the grain-filling stage of crops and even in the next crops. The solitary application of inorganic fertilizers was found inefficient in increasing the crop yield compared to the combined application of organic and inorganic fertilizers [7,12]. Organic fertilizers have more potential in improving crops yield as they contain a high amount of most of the essential plant nutrients (Table 6) and [9,14]. As the C/N ratio of the organic fertilizer was low, the mineralization was faster compared to other organic materials [8]. Thus, the nutrients that were released from the organic fertilizer favored higher nutrient availability and nutrient uptake which resulted in greater source accumulation and efficient translocation of photosynthates into sink, hence, a higher grain yield was obtained. Various reports have suggested the presence of humic substances in VC; the presence of humic acid fraction in an organic amendment makes it agronomically efficient and ecofriendly [21]. Our results revealed that the application of organic and inorganic fertilizer exhibited the maximum straw and biological yield (Table 1). These results are well corroborated with the findings of Bejbaruah et al. [22] and Nowshin et al. [23] who found significant effects of combined application of organic manures and chemical fertilizers on straw yield and biological yield of rice.

### 3.2. Nutrient Uptake by Rice

An interesting observation is that the nutrient uptake by rice was significantly lower with the solitary application of inorganic fertilizers, but the combined application of organic and inorganic fertilizers enhanced the nutrient uptake (Table 2). It further endorses that organic fertilizer is a rich source of nutrients and might contribute to increasing crop yields. The significant linear relationships between uptake of N–P, P–K, and N–K by rice (Figure 1) indicates that except for the control treatment, the nutrient supply in all other treatments were comparatively balanced, however, the amount might vary in different treatments which will ultimately result in variable yields. Moreover, the significant linear relationship between N:K and P:K in rice under different treatments showed that the treatments had a significant effect on the uptake of these nutrients (Figure 2). The N:K and P:K ratios in the present study is endorsed by the study that was conducted by Saleque et al. [6] where they reported an average P/K and N/K ratio of 0.18 and 2.3, respectively. The different ratios of N, P, and K contents in rice plants under different treatments except for the control confirmed the presence of these nutrients in an amount that can satisfy the requirements for the normal growth and development of rice unless other factors are limiting. Many reports showed that different organic fertilizers/composts are the source of N, P, and K that can improve soil fertility and increase the uptake of these nutrients in grain crops [9,19,24]. The continuous use of organic fertilizer along with inorganic fertilizer increased the nutrient uptake and nutrient use efficiency of major nutrients than did the inorganic fertilizers alone [25]. The nutrient uptake is a key component of fertilizer management. The higher amount of nutrient uptake not only maximizes the yield, but also increases the nutrient-use efficiency and, thus, reduces the potential for groundwater pollution. Nitrogen, phosphorus, and potassium are essential for plant growth. The organic fertilizers that were used in the present study (poultry manure, vermicompost) were enriched with different macro- and micro-nutrients especially N, P, and K. The total amount of N, P, and K were high enough and a significant amount might become available from microbial decomposition over the growing season of crops. Rice may uptake a high amount of these nutrients, even over their requirements for normal growth and development, which is normally treated as luxury consumption of nutrients. The uptake of nitrogen by rice was high (Table 2), which may favor the higher uptake of P and K. Nitrogen can increase P uptake and its concentration in plants by increasing root and shoot growth. In most agricultural soils, NO_3_^−^-N is the most usual form which may exceed cation uptake, and, in the mean-time, OH^−^ and HCO_3_^−^ may be released from the roots to the soils [26]. This increases the pH in the rhizosphere and, hence, would promote P availability which, in turn, influences the P uptake by plants.

### 3.3. Nutrient Use Efficiency

All types of use efficiencies of N, P, and K were found within the normal range and, in some cases, were found to be high (Figure 3). In rice cultivation nitrogen use efficiency in terms of agronomic and physiological efficiency were found 14–34, and 34–52, respectively, and in terms of recovery efficiency, it was 33–76% [27]. It was reported that the agronomic efficiencies of N, P, and K in rice were 16–23, 71–101 and 16–36 kg grain kg^−1^ N applied, respectively, and that the recovery efficiencies of these nutrients were 66–117, 48–62, and 24–63%, respectively [7]. The AE of N, P, and K in maize were 50, 48, and 40 kg grain kg^−1^ N applied, respectively, and the RE of these nutrients were 89, 68, and 179%, respectively, as reported by Syafruddin et al. [28]. Higher efficiencies in nutrient use are attributed to the better running of crop and nutrient management practices.

### 3.4. Soil Organic Carbon and Total Carbon

Compared to the control treatment, the organic carbon content in the soil was increased by 10% when PM and VC were applied at the rate of 5 t ha^−1^. Due to the high temperature and humid conditions in our tropical and sub-tropical climatic regions, microbial decomposition of organic materials in soils is very fast and, therefore, the resultant effect is a small increase in carbon content in soils even though farmers apply high amounts of organic matter. However, such a small increase in soil carbon might have a positive effect in increasing the soil carbon stock and thereby reducing the CO_2_ concentration in the atmosphere. Liu et al. [29] reported that animal manure is more effective in building soil C than straw is, possibly due to the presence of more humified and recalcitrant C forms in animal manure as compared to straw. Moreover, manure is more resistant to microbial decomposition than plant residues, thus, for the same C input, C storage is higher with manure application than with plant residues [30]. These results are well corroborated with the findings of Alam et al. [8] who found that the C storage is higher with manure application than with plant residues. The role of the soil microbial communities on biogeochemical processes is influenced by the addition of different organic and inorganic fertilizers in soils. The addition of organic manure increases the growth and activity of soil microbes which revealed a strong relationship between the microbial functioning and the biomass C increase and thus the C content increased more than the initial C content in the soil [31,32].

### 3.5. Total and Available Nitrogen Content in Soil

As VC and PM are the rich sources of nitrogen, both available (ammonium and nitrate nitrogen) and total nitrogen were, therefore, found to be significantly higher in these treatments compared to the control as well as the RD and FP treatments (Table 3). Significant variations in the released ammonium and nitrate nitrogen among the different organic materials and fertilizer treatments were related with the potentiality of mineralization of those added inputs. The factors which control the mineralization of organic matter are the composition or the quality of the residues that are added, soil temperature and water content, drying and rewetting events, soil biota, and soil characteristics [33,34]. The C/N ratios of the organic materials that were used in this experiment were different (Table 4), and thus, there were different mineralization rates. It was reported that organic matter having higher C/N ratios exhibited slower rates of mineralization [12]. During the collection of soil samples at crop harvest, the field was submerged, hence the ammonium contents were higher compared to nitrate nitrogen. Reddy and Kessel [35] also reported that drained and aerated soils contained a higher amount of NO_3_^−^–N, while submerged soils contained a higher amount NH_4_^+^–N. While the mineralization process is slower in wetland soils due to the less efficient and incomplete decomposition of organic matter, NH_4_^+^–N steadily accumulates in the soils and results in higher amounts of NH_4_^+^–N [36]. Rahman [37] reported that NH_4_^+^–N contains a positive charge, and it is attracted by negatively charged clay and humus which, thus, prevents its downward movement from the soil systems. On the other hand, nitrate is a negatively charged ion which is why it cannot be attached to soil particles or soil OM similar to NH_4_^+^. Moreover, NO_3_^−^–N is water soluble and hence is subject to rapid leaching below the crop root zone. As such, the nitrate contents in the top-soils are low. These results are well corroborated with the findings of Rahman [37] who found that the average NH_4_^+^–N in top 5 cm soils was 10 mg kg^−1^, while in 20–30 cm soil depth it was 3 mg kg^−1^. In contrast, the average NO_3_^−^–N in top 5 cm soils was 3.2 mg kg^−1^ and in the 20–30 cm soil depth it was 5 mg kg^−1^.

### 3.6. Physical and Chemical Properties of Post-Harvest Soils

The incorporation of different organic matters significantly increased the physical and chemical properties of soils after crop harvest (Table 4). The changes in the soil bulk density through organic amendments depends on the soil texture. The bulk density of coarse-textured soils are generally higher, while they are comparatively lower in clay loam soils. Therefore, lowering the bulk density due to the application of different organic materials will be more pronounced in sandy soils than in clay loam soils. The addition of organic materials increases soil macropores and deceases meso- and micro-pores, which ultimately contribute to lower soil bulk density. Though the change in soil bulk density in the current study was not much, it gave a positive sign of importance or influence of organic materials on the soil bulk density. The present results well corroborated with the findings of Brar et al. [38] and Brown and Cotton [39] who reported the lowering of the soil bulk density with the application of different organic fertilizer in soils. Rahman et al. [12] also found that organic materials significantly decreased the soil bulk density compared to the control and and inorganic fertilizer treatments. Organic matter is useful for the formation of soil aggregates. Hence, the application of different OM causes a relatively loose structure in the surface layer of the field soil and increase the pore space and thus decreases the bulk density of the soil to favorable levels. Poultry manure was found the most efficient in increasing soil pH to 6.25, which can be rated as slightly acidic (Table 4). This is due to the higher calcium contents in poultry diet which is attributed to poultry manure. Therefore, PM was found to increase soil pH, which was reported by many researchers [7,12,40,41]. Organic matter improved the soil pH status by increasing the soil buffering capacity [40]. Organic fertilizers increase the cation exchange capacity, which contributes to a high base saturation of the soil. As the base saturation increases, the relative amount of acid cations neutralizes. Organic matter, which derived from various sources of organic materials, is a rich pool of supplying essential plant nutrients to the soil [42]. The nutrient availability in the soil is basically reliant on its better physicochemical and biological properties. The application of organic manure enhances the chemical properties of soil, for example, it increases OC, N, P, K, Ca, Mg, and S contents in soil [4,43]. The integrated organic and inorganic fertilizer treatment improved the soil structure and hence increased the macro- and micro-nutrient availability, resulting in enhanced crops yield [44]. Sultana et al. [9] stated that integrated treatment (organic and inorganic) gave higher values for soil N, P, K, and S contents whereas the sole inorganic treatment gave significantly lower values for those nutrients. In fact, the different organic manure increased the bacterial population in the soil, which may enhance the fertility status of soil [45]. Favorable soil pH and nutrient availability conditions also enhance the microbial activity in the soil which might cause the increased availability of nutrients. It was observed that the application of VC and PM at the rate of 2.5 t ha^−1^ contributed to higher amounts of phosphorus (P) (5.87 and 5.79 mg kg^−1^ respectively) to the soils compared to the rate of 5 t ha^−1^ (Table 4). It can be explained in such a way that although the total P is higher with the higher rates of organic fertilizer application, its availability depends on the mineralization of these fertilizers which is a slow process where about 2–3% is mineralized in a year. Moreover, in the case of lower rates of organic fertilizers (VC and PM were applied at the rate of 2.5 t ha^−1^) higher amounts of inorganic P fertilizer (75% of the RD) were applied which might have contributed to a higher availability of P in soils. It was reported that soil pH is important in controlling different available forms of P, as well as precipitation-dissolution and adsorption-desorption reactions, and thus P solubility and availability to plants [46]. Our results showed that soil pH under different organic treatments increased significantly in a favorable range for nutrient availability in soil; it might have a significant positive impact on the nutrient availability. The improvement of soil pH that led to the solubilization of inorganic P is also reported by Whalen et al. [47]. The higher sulphur content in soil could be attributed to a greater mineralization of organic sulphur and release of SO_4_^2−^ ions on its gradual oxidation. Our results were also supported by many other researchers [13,48,49] who reported higher organic carbon, N, P, K, Ca, Mg, and S contents in post-harvest soils due to integrated organic and inorganic fertilizer treatment. It has also been reported that, in addition to the macro-nutrients, organic matter also contains micro-nutrients [44,50], which may promote and maintain the sustainable nutrients supply to the soil.

### 3.7. Soil Organic Carbon Stock, Total Carbon Stock, and Organic Carbon Sequestration in Post-Harvest Soil

The treatments did not show any significant variation in organic carbon stock, total carbon stock, and organic carbon sequestration in post-harvest soils (Table 5). However, compared to the control and RD treatments, small increase in organic carbon stock, total carbon stock and carbon sequestration in soils were due to the addition of poultry manure and vermi-compost at the rate of 5 t ha^−1^. This small increase in the soil organic carbon (SOC) stock may create great impacts in reducing the atmospheric C concentration. Less energy is produced during anaerobic respiration compared to aerobic respiration, and therefore, incompletely decomposed organic materials accumulate in wetland paddy soils resulting in increased C sequestration with organic amendments [36]. The results of the present study are consistent with Rahman et al. [12] who reported that C sequestration in the post-harvest soil increased significantly with the application of organic fertilizers, and, among the treatments, the highest C sequestration was found in the PM application. It was reported that animal manure was more effective in building SOC than straw, possibly due to the presence of more humified and recalcitrant C forms in animal manure as compared to straw [29]. Besides, animal manure is more resistant to microbial decomposition than plant residue; consequently, for the same C input, C storage is higher with animal manure application than with plant residues. It was also reported that the application of different composted manures resulted in a significant amount of C sequestration in the soil, which increased agronomic, physiological, and recovery efficiencies of N, P, and K [7,51]. Therefore, it was evident that C sequestration is essential to increase the use efficiencies of different nutrients.

## 4. Materials and Methods

### 4.1. Experimental Site

The study was conducted at the village Tuk under Kapasia upazila of Gazipur district as well as in the Laboratory of the Department of Soil Science, Bangabandhu Sheikh Mujibur Rahman Agricultural University (BSMRAU), Gazipur 1706, Bangladesh during the period from December 2019 to November 2020. The village has been declared as BSMRAU Technology village by the Bangabandhu Sheikh Mujibur Rahman Agricultural University, Gazipur. The site is located at 24.15° N latitude and 90.37° E longitude with an elevation of 8.2 m from sea level. The experimental area experiences a subtropical climate. It is characterized by comparatively high rainfall, high humidity, high temperature, and a relatively long-day period during April to September while it has scanty rainfall, low humidity, low temperature, and a short-day period during October to March. The latter period is favorable for the growing of dry season rice (*Oryza sativa* L.). The study area is non-saline and belongs to the agro-ecological zone of Old Brahmaputra Floodplain. The soil texture is clay loam under the Sonatala series of Inceptisols order. The overall soil fertility of the experimental field was low to medium. The bulk density of the initial soil was 1.33 g cm^−3^, having field capacity 39.4%, wilting point 20.9%, available moisture content 5%, pH 5.5, organic C 0.85%, total N 0.08%, available phosphorus (P) 2.5 mg kg^−1^, available sulfur (S) 3.0 mg kg^−1^, and exchangeable potassium (K) 0.3 c-mol kg^−1^ soil.

### 4.2. Experimental Treatments and Design

The experiment was designed with seven treatments comprising of (1) zero input control, (2) recommended inorganic fertilizers (RD), (3) poultry manure (PM) (5 t/ha) + 50% RD, (4) PM (2.5 t/ha) + 75% RD, (5) vermicompost (VC) (5 t/ha) + 50% RD, (6) VC (2.5 t/ha) + 75% RD, and (7) farmers’ practice (FP) with three replications that were laid out in a randomized complete block design. The RD of N, P, and K for BRRI dhan29 were 146, 39, and 80 kg ha^−1^, respectively. The unit plot size was 40 m^2^ and the plots were separated from each other by 30 cm bands. The treatments were randomly distributed within each block.

### 4.3. Fertilizer Application, Transplanting, and Intercultural Operations

Poultry manure (PM) and vermicompost (VC) was applied to experimental plots 7 days before the transplanting of rice seedlings. The organic materials were analyzed for moisture, C, N, P, and K contents (Table 6). A soil test-based fertilizer dose of P and K were used as triple super phosphate (TSP) and muriate of potash (MoP), respectively [10]. The recommended doses of N, P, and K of rice were 146, 39, and 80 kg ha^−1^, respectively. The whole amounts of TSP and MoP were applied at the time of final land preparation. Nitrogen as urea was applied in three equal parts in plots 15 days after transplanting (DAT), at maximum tillering stage, and at booting stage of rice. The experiment was conducted using the popular rice variety BRRI dhan29 as a test crop. Seedlings that were 30 days old of BRRI dhan29 were transplanted on January 7. The plant spacing was 20 cm × 20 cm and two seedlings were transplanted in each hill. After the transplanting of rice, 5–6 cm standing water was maintained in each plot throughout the growing period. Weeds were removed by up-rooting from the field three times during the period of the experiment. There was no infestation of pests and diseases in the field and, therefore, no measures were required for controlling pests and diseases.

### 4.4. Harvesting and Data Collection

The rice was harvested at full maturity on May 15. The harvested rice of each plot was bundled separately and the yield data was recorded as kilogram per 40 m^2^ plot and then extrapolated to t ha^−1^. The rice grain yield was recorded on 14% moisture basis. The collected grain and straw samples of rice from each plot were air-dried in the room condition first and then oven-dried at 65 °C for about 24 h until a constant weight was obtained after which they were ground by a grinding machine. Later the ground samples were sieved through a 20-mesh sieve. The rice samples (both grain and straw) were analyzed for N, P, and K. The total nitrogen in the plant samples was determined using Kjeldhal systems [52], whereas the total P and K were determined by the acid digestion method [53,54].

The soil samples after the harvesting of the rice were also collected and analyzed for residual soil nutrients. After harvesting of the crop, the soil samples were collected from each plot at 0–15 cm depth and analyzed for bulk density pH, organic carbon (OC), total carbon (TC), and residual nutrients. The bulk density was determined by a core sampler method [55]. The soil pH was determined by a glass electrode pH meter method with soil water ratio 1:2.5 [56]. The organic carbon was determined by Walkley-Black method [57], the total carbon was determined by dry combustion method using C-N analyzer. The total nitrogen was determined by Kjeldhal systems [52], the available nitrogen [nitrate (NO_3_^−^)-N plus ammonium (NH_4_^+^)-N] was determined by the method that was given by Keeney and Nelson [58], the available P was determined by Olsen’s method [59], and sulphur was determined by Turbidimetric method. Potassium, calcium, and magnesium were determined by ammonium acetate extraction method [60].

The recovery efficiency, agronomic efficiency (AE), and physiological efficiency (PE) of nutrients for rice were calculated by using the following formula [27].

RE (%) = [(Nup_f_ − Nup_0_)/Nap] × 100

AE (kg grain kg^−1^ nutrient applied) = (Y_f_ − Y_0_)/N_ap_

PE (kg grain kg^−1^ nutrient uptake) = (Y_f_ − Y_0_)/(N_upf_ − N_up0_)

where,

Nup_f_ = Nutrient uptake from fertilizer applied plot or treatment (kg ha^−1^)

Nup_0_ = nutrient uptake from control plot (kg ha^−1^)

Nap = Amount of nutrient applied (kg ha^−1^)

Y_f_ = Grain yield of fertilized plot (kg ha^−1^)

Y_0_ = Grain yield of control plot (kg ha^−1^)

The nutrient applied is the sum of the individual nutrient that is added to the soil using poultry manure, vermicompost, and inorganic fertilizer as per the treatments.

The carbon stock and its sequestration in 15 cm soil under the different treatments were calculated using the following equations that were given by Rahman et al. [12].

Carbon stock (t ha^−1^) = Carbon concentration (%) × bulk density (g cm^−3^) × depth (cm)

Carbon sequestration (t ha^−1^) = Final C stock (t ha^−1^) − Initial C stock (t ha^−1^)

To calculate the initial carbon stock, the soil carbon concentration and the bulk density were determined by collecting soil samples before starting the experiment where the values were 0.85% and 1.33 g cm^−3^, respectively.

### 4.5. Statistical Analysis

SPSS version 12.0 statistical software (SPSS Inc., Chicago, IL, USA) was used to analyze the data. A one-way analysis of variance (ANOVA) and univariate analysis were performed. The means were separated by least significant difference (LSD (0.05)).

## 5. Conclusions

The imbalanced use of inorganic fertilizers without organic fertilizers has led to the deterioration of soil health. Organic manure contains essential plant nutrients that can be reused for crop production. We examined the effects of integrated nutrient management on rice yield, nutrient use efficiency, soil fertility, and carbon (C) sequestration in cultivated land. The combined application of organic and inorganic fertilizers significantly influenced the rice yield, nutrient uptake, and their subsequent use efficiencies, contents of organic carbon, total carbon, total nitrogen, ammonium nitrogen, nitrate nitrogen, soil pH, phosphorus, potassium, sulfur, calcium, magnesium, and C sequestration in post-harvest soil. Therefore, it is concluded that integrated nutrient management is crucial for sustainable crop production.

## Figures and Tables

**Table 1:** The yield of rice under different treatments of organic manures and inorganic fertilizers.

Treatments	Grain Yield (t ha^−1^)	Straw Yield (t ha^−1^)	Biological Yield (t ha^−1^)
Control	3.63 d	4.00 d	7.6 d
RD	5.33 c	6.41 c	11.7 c
PM5 + 50% RD	5.86 ab	6.81 bc	12.7 b
PM2.5 + 75% RD	6.16 a	7.24 ab	13.4 a
VC5 + 50% RD	5.60 bc	6.76 c	12.4 b
VC2.5 + 75% RD	6.27 a	7.35 a	13.6 a
FP	5.42 c	6.66 c	12.1 bc
S.E. (±)	0.19	0.21	0.61
CV (%)	4.46	4.00	5.1

Means followed by the uncommon letter (s) are significantly different from each other at the 5% level of significance by DMRT. Note: RD = recommended inorganic fertilizers; PM = poultry manure; VC = vermicompost; FP = farmer’s practice.

**Table 2:** The nutrient uptake by rice under different treatments of organic manures and inorganic fertilizers.

Treatments	Nutrient Uptake (kg ha^−1^)		
	N	P	K
Control	95.41 e	16.65 d	82.87 g
RD	145.16 d	25.41 bc	104.69 f
PM5 + 50% RD	157.34 bc	29.57 a	109.14 e
PM2.5 + 75% RD	166.06 ab	27.14 b	115.50 d
VC5 + 50% RD	152.67 cd	24.14 c	135.46 b
VC2.5 + 75% RD	168.86 a	26.70 b	145.97 a
FP	148.85 cd	25.46 bc	130.52 c
S.E. (±)	4.95	0.87	1.57
CV (%)	4.10	4.24	1.63

Means followed by the uncommon letter (s) are significantly different from each other at the 5% level of significance by DMRT. Note: RD = recommended inorganic fertilizers; PM = poultry manure; VC = vermicompost; FP = farmer’s practice.

**Table 3:** Carbon and nitrogen status after rice harvest under different treatments of organic manures and inorganic fertilizers.

Treatments	Organic Carbon (%)	Total Carbon (%)	Total Nitrogen (%)	Ammonium Nitrogen (mg kg^−1^)	Nitrate Nitrogen (mg kg^−1^)
Control	0.95	1.00 ab	0.077 c	3.69 c	1.56 b
RD	0.96	1.02 ab	0.073 c	7.45 ab	2.74 ab
PM5 + 50% RD	1.05	1.18 a	0.124 a	9.21 ab	3.55 a
PM2.5 + 75% RD	0.99	1.03 ab	0.086 bc	9.29 a	2.70 ab
VC5 + 50% RD	1.03	1.14 a	0.109 ab	8.06 ab	2.76 ab
VC2.5 + 75% RD	0.95	1.02 ab	0.099 abc	8.32 ab	1.92 b
FP	0.92	0.94 b	0.085 bc	6.99 b	1.61 b
S.E. (±)	0.13	0.14	0.012	1.04	0.56
CV (%)	6.17	5.39	6.01	6.83	8.83

Means followed by the uncommon letter (s) are significantly different from each other at the 5% level of significance by DMRT. Note: RD = recommended inorganic fertilizers; PM = poultry manure; VC = vermicompost; FP = farmer’s practice.

**Table 4:** Soil physical and chemical properties after rice harvest.

Treatments	BD (g cm^−3^)	Soil pH	P (mg kg^−1^)	S (mg kg^−1^)	K (c-mol kg^−1^)	Ca (c-mol kg^−1^)	Mg (c-mol kg^−1^)
Control	1.32 ab	5.47 c	2.69 c	2.89 c	0.12 d	1.88 e	0.24 d
RD	1.35 a	5.63 c	3.79 bc	5.24 b	0.21 c	2.27 de	0.37 d
PM5 + 50% RD	1.18 e	6.25 a	5.66 ab	4.75 bc	0.28 ab	3.80 b	0.57 c
PM2.5 + 75% RD	1.23 cde	5.72 c	5.87 a	4.77 bc	0.23 bc	3.06 c	0.82 b
VC5 + 50% RDF	1.21 de	6.07 ab	4.02 abc	8.85 a	0.33 a	5.07 a	0.61 c
VC2.5 + 75% RD	1.26 bcd	5.61 c	5.79 a	6.42 b	0.23 bc	2.70 cd	1.14 a
FP	1.28 bc	5.77 bc	3.51 c	6.07 b	0.13 d	2.90 cd	0.38 d
S.E. (±)	0.025	0.15	0.87	0.93	0.03	0.32	0.07
CV (%)	2.43	3.10	13.99	10.40	7.55	12.49	8.48

Means followed by the uncommon letter (s) are significantly different from each other at the 5% level of significance by DMRT. Note: RD = recommended inorganic fertilizers; PM = poultry manure; VC = vermicompost; FP = farmer’s practice; BD = bulk density.

**Table 5:** Effects of poultry manure, vermicompost, and inorganic fertilizers on bulk density, carbon stock, and carbon sequestration in post-harvest soil.

Treatments	OC Stock (t ha^−1^)	TC Stock (t ha^−1^)	OC Seq. (t ha^−1^)
Control	17.44	18.43	0.49
RD	18.26	19.84	1.31
PM5 + 50% RD	18.70	20.81	1.75
PM2.5 + 75% RD	17.74	16.71	0.79
VC5 + 50% RD	18.59	20.76	1.65
VC2.5 + 75% RD	17.98	20.60	1.03
FP	17.58	19.61	0.63
S.E. (±)	2.08	2.44	2.08
CV (%)	14.15	15.31	33.87

Note: RD = recommended inorganic fertilizers; PM = poultry manure; VC = vermicompost; FP = farmer’s practice; OC = organic carbon; TC = total carbon; OC seq. = organic carbon sequestration.

**Table 6:** Moisture, carbon, and nutrient content of poultry manure and vermicompost.

Sample	Moisture (%)	Carbon (%)	Nutrient Content (%)		
			N	P	K
Poultry manure	35	16.82	1.33	0.75	0.85
Vermicompost	45	18.21	1.57	1.25	2.00

## Data Availability

The data that support the findings of this study are available from the corresponding author upon reasonable request.

## Abbreviations

C sequestration	Carbon sequestration
RD	Recommended inorganic fertilizers
PM	Poultry manure
VC	Vermicompost
FP	Farmers’ practice
N	Nitrogen
P	Phosphorus
K	Potassium
Ca	Calcium
Mg	Magnesium
S	Sulfur
INM	Integrated nutrient management
OM	Organic matter
CO_2_	Carbon dioxide
OC	Organic carbon
TC	Total carbon
BRRI	Bangladesh Rice Research Institute
TSP	Triple super phosphate
MoP	Muriate of potash
DAT	Days after transplanting
RE	Recovery efficiency
AE	Agronomic efficiency
PE	Physiological efficiency
Total N	Total nitrogen
SOC stock	Soil organic carbon stock
OC seq.	Organic carbon sequestration
NO_3_^−^-N	Nitrate nitrogen
OH−	Hydroxide
HCO_3_^−^	Bicarbonate
NH_4_^+^–N	Ammonium-nitrogen
NH_4_^+^	Ammonium ion
